# The Ebola Vaccine, Iatrogenic Injuries, and Legal Liability

**DOI:** 10.1371/journal.pmed.1001911

**Published:** 2015-12-01

**Authors:** Amir Attaran, Kumanan Wilson

**Affiliations:** 1 Institute of Population Health, Faculty of Law, Faculty of Medicine, University of Ottawa, Ottawa, Ontario, Canada; 2 Department of Medicine, University of Ottawa, Ottawa, Ontario, Canada; 3 Ottawa Hospital Research Institute, Ottawa, Ontario, Canada

## Abstract

Amir Attaran and Kumanan Wilson propose a compensation system for vaccine injuries, based on no-fault principles, to ensure that recipients of Ebola vaccines are fairly compensated in cases of iatrogenic harm.

**Summary Points**

- The development and eventual deployment of an Ebola vaccine was delayed for various technical and financial reasons, but with the apparent success of a vaccine candidate in a recently reported clinical trial, an urgent problem is the lack of any system to protect vaccine firms from the risks of legal liability caused by vaccine-related injuries.
- Without indemnity or security against the legal risks, vaccine firms are less likely to engage in research and development of vaccines, particularly for rare diseases of poor countries such as Ebola.
- WHO’s traditional method of mitigating the legal risks through indemnification agreements with countries appears too slow to implement in urgent pandemic situations. Also, the enforceability of any WHO-backed legal agreement is placed in doubt because the United Nations has the option to claim immunity from lawsuits.
- Creating a compensation system for vaccine injuries, based on no-fault principles and, most likely, overseen by the World Bank, could address the liability concerns and facilitate getting novel vaccines into clinical trials and to the market. This system would also ensure that recipients of these vaccines are fairly compensated in the rare instances that they are harmed.

Making vaccines is a risky, oftentimes unenviable business. Vaccines are administered to healthy people who tend to be unforgiving if an adverse side effect or injury subsequently develops. The risk of being sued, even when a vaccine supplier follows best practices, combined with growing anti-vaccination sentiment, creates a climate that is not conducive to vaccine innovation. The dissuasive effect of litigation risk and legal liability is heightened both for vaccines aimed at diseases of poor countries, for which the financial inducements are weak anyway [1,2], and vaccines for public health emergencies, which are developed in accelerated clinical trials that may lack the statistical power or detailed follow-up necessary to detect rare adverse effects. Yet, as the West African Ebola outbreak demonstrates, the world can ill afford not to have vaccines against diseases of poverty in emergency situations [1]. Several reasons exist for not having a vaccine available, relating to the biology of the virus and the epidemiological challenges pertaining to evaluating a vaccine for a rare disease. However, financial incentives and disincentives for vaccine manufactures to invest in vaccine trials for rare diseases in resource-poor countries also need to be considered. We argue that, as one part of a comprehensive plan to promote vaccine development, there needs to be a plan to lessen the risks of litigation and liability to remove disincentives for these vaccines to be developed and later deployed. As others point out, no satisfactory plan now exists [3].

In the past, worries about litigation and liability have delayed the availability of vaccines, even as other parts of the emergency response have been hastened. For example, during the 2009 H1N1 influenza pandemic, WHO reduced the process of prequalifying vaccines for safety and efficacy from the usual 12–24 months to as little as one day—but could not accelerate the legal issues similarly [4]. In Africa, the typical time taken from a country expressing interest to WHO about receiving donated vaccine, and signing the “letter of agreement” containing the liability arrangements, was about 100 days [5]. Progress was stalled by some countries lacking resources to interpret the liability issues that this vaccine raised. Liability holdups probably explain why some countries received vaccine after the pandemic’s peak had passed [4].

Post-H1N1, WHO recommended to “prepare in advance and maintain a framework…to expedite legal agreements during future pandemic events” [5]. That framework, however, never came into being, and so in this paper we review three options that have currency now, as the interim results of a clinical trial have demonstrated 100% protective efficacy for an Ebola vaccine [6]. The recognized options include the following: (1) the country experiencing the public health emergency can indemnify the vaccine supplier, or (2) the United Nations can use its immunity from lawsuit to shield the vaccine supplier. But we believe that both of these are surpassed by a third, superior option, in which (3) the international community can establish a no-fault compensation fund to fairly redress vaccine injuries. The three options are discussed more fully here.

## Government Indemnity

In developed countries, it is often the case that governments advance the public goods of vaccination by assuming liability for vaccine injuries on a “no fault” basis (i.e. without a finding of wrongdoing) [7]. This requires a complex legislative scheme to resolve injury claims fairly outside of the courts. For example, in the United States, laws create an injury compensation fund and “vaccine court,” supported by a tax on vaccine sales, and shield vaccine manufacturers from lawsuits [8]. The system creates indemnity for select adult and childhood vaccines and for vaccines related to a declared public health emergency, except in the event of willful misconduct [9].

However, nearly all developing countries, and some developed ones (e.g., Canada), lack such national laws. Since authoring complex legislation during a pandemic is between difficult and infeasible, these governments can instead use interim, ad hoc arrangements in contract law to assume the risks faced by vaccine suppliers. These are commonly known as indemnity, or “hold harmless,” agreements.

Contractual agreements are by nature bilateral: there needs to be one for each manufacturer or supplier needing indemnification, although these could, if desired, conform to a template. The agreement can take the form of a stand-alone indemnity contract, or an indemnity clause can be included in the contract of sale. During the H1N1 pandemic, many countries that received vaccine donations relied on a WHO-drawn template, although some made independent arrangements. Regardless, the general formula is to provide the vaccine supplier a legally binding assurance that the government assumes all liability for vaccine-related injury, so long as the supplier complies with current good manufacturing practices and agreed-on specifications.

The main limitation of the contractual approach is that while it can provide the vaccine supplier indemnity from liability for wrongdoing, it cannot make the supplier immune from being sued. Therefore, a supplier may still have to appear before the courts and defend litigation, which even if unsuccessful is a negative incentive for a vaccine manufacturer to invest in novel vaccines. This was true in a recent Canadian case in which, despite an indemnity agreement for the H1N1 influenza vaccine, GlaxoSmithKline was sued (it defended itself successfully) [10]. Indemnity agreements, therefore, do not wholly remove the risks of litigation, and if the court finds that a vaccine supplier behaved negligently or fraudulently, this may even permit the indemnity agreement to be bypassed or voided, depending on its wording and circumstances [11].

## United Nations Immunity

The alternative to indemnity from liability is immunity from suit. The property and assets of the United Nations, including WHO, normally enjoy “immunity from every form of legal process” [12]. Immunity, however, is not meant to create impunity, and international law also obliges the UN to “make provisions for [alternative] modes of settlement,” usually involving arbitration [11]. Thus, when the UN acts on public health it is shielded from lawsuits, but the quid pro quo is that it must take responsibility for any harm it causes through other means.

The UN’s immunity was used to good advantage as a liability shield during the H1N1 pandemic. Some vaccines were passed from the manufacturer, to WHO, to the recipient country, in that order [4]. The pass-through transferred certain legal liabilities to WHO and immunized them from lawsuit, which is clearly an elegant solution from the supplier’s perspective. But unfortunately, as demonstrated by the events of the 2010 earthquake in Haiti, where UN peacekeepers introduced a virulent, Asian strain of cholera bacteria to that country, resulting in about 7,500 reported deaths [13], an immunity-based approach can be problematic for the recipient. Briefly, the UN has failed to honor its commitment under a signed agreement with Haiti’s government (a Status of Forces Agreement signed in 2004) to establish a standing claims commission to adjudicate the cholera victims’ claims fairly [14–17] and also invoked its immunity from lawsuit to have the victims’ claims thrown out of court, to date successfully [18,19]—thus effectively denying the victims compensation for their injuries caused by the actions of UN peacekeepers.

A consequence of these unprecedented actions is that developing country governments, which were already slow during the H1N1 pandemic to enter into legal agreements with WHO, may now have another reason not to enter into agreements in which the UN agrees to compensate victims of vaccine injuries. In such circumstances, vaccine suppliers may have to contend with frustrated victims and the courts who might turn and impose liability on them. These factors make it difficult, perhaps impossible, for WHO to obtain the consent from vaccine suppliers and vaccine recipients to make a legal template such as was used for the H1N1 pandemic workable again. Some other solution therefore appears needed.

## No-Fault Compensation

The third and, from our perspective, preferred option is for the international community to establish a no-fault compensation fund for novel vaccines released on an emergency basis to low-income countries.

No-fault compensation funds are in widespread use in highly developed vaccine markets: at least 19 jurisdictions around the world have such a system [20,21]. Their purpose is to provide rapid, equitable compensation for injuries causally related to vaccination, without resort to damaging litigation. For example, in the 1980s, US litigation surrounding the diphtheria, tetanus, and whole cell pertussis vaccine caused some manufacturers to cease production, leading to shortages, rationing, and skyrocketing prices [22]. The resulting system, which became operational in 1988, pegged compensation to a clinical and administrative determination of vaccine-related injury, and not a judicial finding of wrongdoing by the supplier (hence “no-fault”). Funding was obtained from a vaccine excise tax, with payouts limited to conditions found in a “table of injuries” for which there is evidence that vaccination can be causal. Off-table injuries are addressed by a specialized “vaccine court.” All this has helped avoid civil litigation and, for example, shielded vaccine manufacturers from costs associated with civil lawsuits pertaining to autism and vaccines [23]. However, there have been criticisms of the US no-fault approach, including a high rate of off-table determinations, lengthy case timelines and an increasingly adversarial nature to the proceedings [24,25].

There are variations on the US no-fault model. In Québec, there is not a vaccine excise tax or a standing fund to pay compensation, and instead the government uses general revenue to pay damages and the province’s auto insurance scheme to administer awards [20,26]. Adjudication of cases in Québec is conducted by a three-person committee.

A no-fault compensation program for an Ebola vaccine released on an emergency basis would require some adjustments on these models (Table 1). First, given that the vaccine is new, there are not pharmacovigilance data to ascertain the adverse effects and to construct a table of injuries. Instead, assessing causality and setting the quantum of compensation initially would have to be done by expert consensus methods and epidemiological inference [27]. Second, because an excise tax on an Ebola vaccine that is meant for poor people would be an inequitable and regressive way to capitalize a compensation fund, instead it would be fairer if donors from developed countries capitalized that fund directly, for hastening the deployment of vaccines in developing countries without being delayed by worries about litigation and legal liability. Doing so is in the collective interest of security from pandemics.

**Table 1:** Components of a proposed international no-fault compensation program for vaccine injuries in developing countries.

Vaccines Covered	Novel vaccine emergently released to low-income countries and approved by WHO or other stringent regulatory authority on an emergency basis.
Administrative Body	World Bank
Source of Funding	Short term: World Bank Avian and Human Influenza Facility unspent balances.
	Long term: a World Bank mechanism funded by donors and modeled on the Global Facility for Disaster Reduction and Recovery.
Causality assessment	When possible, a table of injuries approach. When not possible, expert review guided by the following principles:
	• biological plausibility as based on expert opinion;
	• temporal relationship between injury and vaccination;
	• logical sequence of cause and effect; and
	• no other probable cause.
Compensable events	Any medical condition satisfying the causality assessment, occurring within the prescribed timeframe (e.g., anaphylaxis should follow vaccination within hours), and that is properly submitted as a claim within one year.
Compensation	To be determined based on available resources.
	Benchmark: the United Kingdom pays £120,000 as a one-off vaccine damage payment [28]. This could be indexed relative to the per capita income of the country where the injury is suffered.
	Hypothetical: the per capita income of Ruritania relative to the United Kingdom is 10%, for compensation of £12,000 (~US$19,000). About 1,000 persons could be compensated using the last-reported residual of the Avian and Human Influenza Facility (US$20 million).

WHO has already proposed that the World Bank take the lead on indemnifying the suppliers of an Ebola vaccine [29]. We agree, and believe that the Bank already has appropriate mechanisms at hand.

First, the recently shuttered World Bank trust fund for avian influenza (its last grant closed in March 2014) could be repurposed in the very short-term to meet the needs of Ebola vaccine liability. At last report, that trust fund contained about US$20 million in unspent donor contributions, which could serve as an initial commitment toward a fund to compensate vaccine injury victims [30]. Given that the same donors concerned with influenza also have reason to worry about Ebola and other disease emergencies, it should be feasible to shift these funds from one pandemic to another, and doing so could be viewed as a transitional measure toward the permanent Global Pandemic Emergency Facility recently proposed by the World Bank [31].

Second, and with a view to future emergencies, the World Bank could develop an international no-fault compensation scheme for vaccine injuries. Drawing on Québec’s approach, insurers could provide coverage with premiums paid for by donor funds. Since the insurance and capital markets have little experience covering vaccine risks, this capacity would have to be incubated by the World Bank, perhaps in the footsteps of its Global Facility for Disaster Reduction and Recovery. This cluster of the Bank and 25 donor countries has arranged various sorts of disaster insurance, such as “catastrophe bonds” to cover risks such as typhoons, earthquakes, or crop failures due to weather. If capital markets are willing to insure those unpredictable, large risks, then certainly the similarly unpredictable but much more contained risk of vaccine injuries could be insured as well.

## Conclusion

The Ebola outbreak has revealed myriad systemic deficiencies in health systems, international institutions, drug development and global readiness for public health emergencies. To this list, one must now add the lack of arrangements for vaccines to be deployed rapidly without hesitation because of liability. Whatever ad hoc arrangements are made should lay a sustainable foundation for future outbreaks and vaccines.

We think it is not too ambitious in the time before an Ebola vaccine is available for routine use to create an international no-fault vaccine injury compensation program. This program could be disbursed upon the advice of an expert panel agreeing as to causality, and funded through the World Bank’s residual influenza trust funds, plus possibly other Ebola-specific contributions. In the intermediate term, such an arrangement should be institutionalized through the World Bank, its access to insurance markets, and its proposed Global Pandemic Emergency Facility. In the long term, countries should be given assistance to devise no-fault compensation systems within their domestic law, so as to obviate the need to manage liability at the international level. This suite of approaches both suffices for Ebola and lays a foundation for future emergently released vaccines directed primarily at resource-poor countries.
